# Evaluation of prosthetic dysfunction in the diagnosis of endocarditis associated to prosthetic pulmonary valve and pulmonary conduit

**DOI:** 10.1016/j.ijcchd.2025.100591

**Published:** 2025-05-13

**Authors:** Andrés Cano Pérez, Larraitz Orive Melero, Jose Félix Larrea Egurbide, Jagoba Larrazábal López, Luis Fernández González, Roberto Blanco Mata, Josune Arriola Meabe, Leire Artiñano Mendizábal, Ane Josune Goikoetxea Agirre, María José Blanco Vidal, Javier Ayala Curiel, Pedro María Montes Orbe

**Affiliations:** a Cardiology Department, Cruces University Hospital, Baracaldo, Spain; b Infectious Diseases Department, Cruces University Hospital, Baracaldo, Spain; c Paediatric Cardiology Department, Cruces University Hospital, Baracaldo, Spain

**Keywords:** Pulmonary valve prosthesis, Right ventricle to pulmonary artery conduit, Transcatheter pulmonary valve, Infective endocarditis, Transtorathic echocardiography, Prosthetic dysfunction

## Abstract

**Introduction:**

The number of cases of infective endocarditis associated to prosthetic pulmonary valves and pulmonary conduits (PPVIE) is likely to increase in the future. Transthoracic echocardiography (TTE) presents challenges in visualizing lesions suggestive of endocarditis in these patients. However, TTE may provide additional findings, such as prosthetic dysfunction, which can guide the diagnosis of this condition. The main objective of this study is to analyze prosthetic pulmonary valve dysfunction as an echocardiographic manifestation of PPVIE.

**Methods:**

All cases of PPVIE (definite and possible, according to the modified Duke criteria) at Cruces University Hospital (Baracaldo, Spain) between January 2014 and July 2024 were included. Prosthetic dysfunction was defined as a peak transvalvular gradient (PTG) ≥25 mmHg for homografts and ≥40 mmHg for prosthetic pulmonary valves and bovine pulmonary conduits (stenosis) and/or pulmonary regurgitation (PR) moderate or severe. Number of cases of prosthetic dysfunction between the PPVIE episode and prior to the episode were compared. We analyzed the mechanisms of prosthetic dysfunction in the PPVIE episode.

**Results:**

14 cases of PPVIE were identified. In cases prior to the PPVIE episode, 42.9 % had prosthetic dysfunction, while in the PPVIE episode, 92.3 % had prosthetic dysfunction. Stenosis was a more frequent cause of valve dysfunction than PR.

**Conclusions:**

Prosthetic dysfunction (due to stenosis or regurgitation) is a relevant finding in the diagnosis of PPVIE in cases where lesions suggestive of endocarditis are not visualised. Although not included in the Duke criteria, stenosis is a more frequent mechanism of dysfunction than PR.

## Highlights

• Cases of prosthetic pulmonary valve endocarditis are expected to rise.
• Its diagnosis by transthoracic echocardiography can be challenging.
• Prosthetic dysfunction is a relevant finding when the diagnosis is uncertain.
• Stenosis is more frequent than pulmonary regurgitation as dysfunction mechanism.

## Graphical abstract

**Figure:**
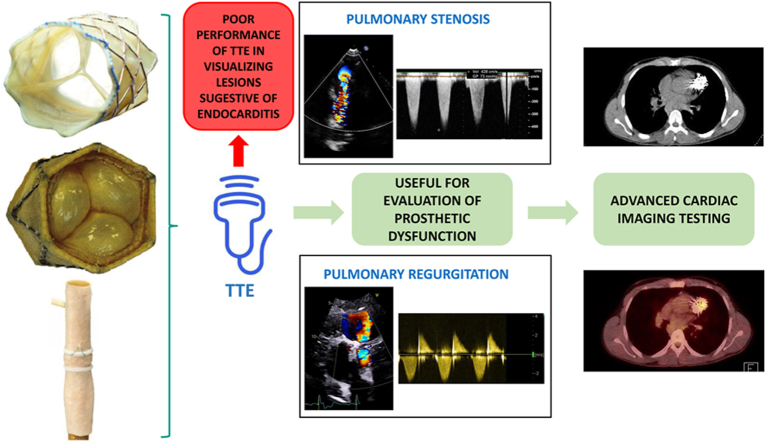

## 1 Introduction

Patients with congenital heart disease (CHD) have experienced a remarkable increase in survival rates over the past decades. Advances in prenatal and postnatal diagnosis, treatment, and follow-up have enabled 95 % of these patients to reach adulthood [1]. However, many of them remain in a palliated state or present residual lesions that will require reinterventions throughout their lives. This situation, combined with the development of surgical techniques and the exponential increase in the implantation of transcatheter devices and valves, has led to a rise in potential complications. Among these complications is infective endocarditis (IE), which occurs at an incidence 27–44 times higher compared to the general population [2].

CHD encompasses a heterogeneous group of conditions, with those affecting the pulmonary valve (PV) or the right ventricular outflow tract (RVOT) being particularly common. Prosthetic pulmonary valves (PPV), whether surgically implanted or transcatheter, and pulmonary conduits (PC) are commonly used in their repair. The introduction of transcatheter pulmonary valve implantation has revolutionized the treatment of these patients and has shown promising outcomes. However, IE has emerged as a major complication associated with these valves, causing significant morbidity and mortality [3].

Transthoracic echocardiography (TTE) is the first-line imaging modality used in the diagnosis of IE [4]. However, prosthetic pulmonary valve or pulmonary conduit-associated infective endocarditis (PPVIE) presents several challenges that complicate its diagnosis using this technique. The anterior position of the RVOT or PC within the thorax hinders adequate visualization. Additionally, anatomy may be altered due to previous corrective surgeries, as the pulmonary prosthesis may be positioned differently from the native valve (especially in the case of PC), or due to the underlying CHD itself. Moreover, PPV and PC may develop calcifications that create acoustic shadowing, leading to imaging artifacts [5].

Nevertheless, TTE remains crucial for assessing hemodynamic impact and valvular dysfunction, which can present as either outflow obstruction or valvular regurgitation. These same findings may also be seen in other conditions, such as thrombosis or valve degeneration. However, in the presence of clinical suspicion of IE, further advanced imaging studies may be conducted to rule out the condition.

Given the expected increase in the incidence of PPVIE, it is essential to expand research efforts to document and describe previously unreported cases. The present study aims to describe the main clinical and microbiological characteristics of all PPVIE cases diagnosed at a tertiary hospital with expertise in CHD between January 2014 and July 2024, as well as to assess the presence of prosthetic dysfunction and its utility as a relevant diagnostic finding.

## 2 Methods

In this single-centre descriptive study, all patients diagnosed with PPVIE at Cruces University Hospital (Vizcaya, Spain) between January 2014 and July 2024 were included. All patients were evaluated by at least one specialist in Infectious Diseases and one specialist in Cardiology with experience in CHD (both pediatric and adult patients).

Cases were identified using the hospital database's electronic search tool (Minimum Basic Data Set – Specialized Care of Cruces University Hospital). Only patients with a possible or confirmed PPVIE diagnosis according to the Duke criteria were selected. Cases diagnosed prior to the latest update of the Duke criteria (2023) [6] were classified as possible or confirmed IE according to the current criteria at the time of diagnosis [7,8]. The date of IE diagnosis was considered the day of the initial medical contact in the PPVIE episode. Early IE was defined as occurring within the first 12 months after prosthesis implantation, while late IE was defined as occurring beyond this period [4]. All clinical and microbiological data were extracted from medical records. Follow-up time was considered as the date of death or the beginning of the present study (July 31, 2024).

Regarding imaging data, it was recorded whether patients had undergone a TTE prior to the IE diagnosis, and reports and images from both the pre-IE TTE and the TTE during the IE episode were reviewed. The degree of pulmonary stenosis (PS) was quantified using the peak pulmonary transvalvular gradient (PTG). The mean gradient was not used as it was not available in all studies prior to the IE episode. Additionally, the degree of pulmonary regurgitation (PR) was quantified using color Doppler and continuous-wave Doppler as absent, mild, moderate, or severe [9]. The presence of major endocarditis-associated lesions was recorded, including vegetations, leaflet rupture or perforation, paravalvular complications (abscess, pseudoaneurysm or fistula), or prosthetic valve dehiscence. The study was classified as compatible with IE if any of these lesions were present [6]. If not, the study was classified as non-diagnostic (whether it was inconclusive or negative). PTG and PR severity were compared between the pre-IE TTE and the TTE during the IE episode.

Prosthetic dysfunction was defined as the presence of a PTG ≥40 mmHg in the case of PPV and bovine PC, and PTG ≥25 mmHg in the case of homografts; or PR greater than mild (moderate or severe), according to the American Society of Echocardiography's recommendations for imaging assessment of cardiac valve prosthesis [9].

Regarding the use of complementary cardiac imaging techniques, it was recorded which techniques followed TTE, both in patients with TTE compatible with IE and in patients with non-diagnostic TTE. Among patients with a non-diagnostic TTE, we analyzed the sensibility of each of the complementary imaging techniques and their overall ability to reclassify these patients as definite IE.

Continuous variables are expressed as median and interquartile range. Categorical variables are expressed as absolute values or percentages.

## 3 Results

### 3.1 Clinical data

A total of 14 cases of PPVIE were identified during the study period in 12 patients. Two patients experienced two episodes of PPVIE during this period. The median age at diagnosis was 21.1 years (11.7–24.1), and 78.6 % were male. The most common CHD was Tetralogy of Fallot (57.1 %). Patients had undergone a median of 2.5 cardiac surgeries (2.0–4.0) and 1 transcatheter intervention (0.0–2.0) involving the PV or RVOT. There were 11 cases of late IE and 3 cases of early IE. Median time between prosthesis implantation and PPVIE diagnosis was 4.6 years (0.9–7.8).

PPVIE occurred in 57.1 % of cases on a PPV, with 35.7 % involving a percutaneous Melody™ valve (Melody™ Transcatheter Pulmonary Valve, Medtronic Inc., Minneapolis, MN, USA) and 21.4 % involving a surgically implanted Injectable BioPulmonic™ valve (BioIntegral Surgical, Inc., Ontario, Canada). The remaining 42.9 % of cases involved PC, with 35.7 % on a Contegra™ (Contegra™ Pulmonary Valved Conduit, Medtronic Inc., Minneapolis, MN, USA) and 7.2 % on a Bioconduit™ (BioIntegral Surgical, Inc., Ontario, Canada).

In four cases, the patient had experienced at least one previous IE episode before the PPVIE episode leading to the inclusion in the present study. Two of these cases involved the same patient, who had two independent PPVIE episodes included in this study (PPVIE on a Melody™ valve in July 2020 of unknown etiology treated conservatively, and PPVIE on a Melody™ valve in October 2021 requiring surgical intervention, with a positive polymerase chain reaction (PCR) test for *Coxiella burnetii* in the explanted material). This patient had also experienced a prior PPVIE episode on a Contegra™ conduit due to *Streptococcus mitis* in February 2017, which was not included in this study as it was managed at another centre. Another patient had two PPVIE episodes of different etiologies involving a Melody™ valve (September 2020 due to *Streptococcus anginosus* and March 2021 due to *Streptococcus mitis*), both included in the study. Lastly, one patient had a previous PPVIE episode due to *Streptococcus mitis* on a Contegra™ conduit in 2013 (prior to the study period). None of the patients had other known risk factors for right-sided IE, such as intracardiac device implantation, immunosuppression, intravenous drug use, or central venous catheterization.

Fever (T^a^ ≥ 38 °C) was present in 64.3 % of cases, mostly accompanied by nonspecific symptoms such as malaise or myalgia. Only two patients presented with severe heart failure symptoms, and one with septic shock. Additional clinical characteristics of the patients are summarized in Table 1.

**Table 1:** Clinical and demographical data.

Gender (Male)			11 (78,6 %)
Age (years)			21,1 (11,6–24,0)
Type of CHD			
	Tetralogy of Fallot		8
	Pulmonary atresia with VSD		2
	PA with intact ventricular septum		1
	Congenital pulmonary stenosis		1
	Complex transposition of great arteries		1
	Truncus arteriosus		1
Previous sternotomies			2,5 (2,0–4,0)
Previous transcatheter interventions on the PV			1,0 (0,0–2,0)
Chronic kidney disease			0
Hepatic failure			0
Chronic respiratory failure or severe lung disease			0
Disabling neurological disease			1
Risk factors of right-sided IE			
	Intracardiac electronic device		0
	Immunodepression		0
	Intravenous drug abuse		0
	Intravenous/central venous catheter		0
Previous IE			4
Type of RV-PA substrate			
	Contegra™		5
	BioConduit™		1
	BioPulmonic™		3
	Melody™		5
		In Contegra™	2
		In homograft	3
Clinical presentation			
	Fever (T^a^≥38°)		9
	Heart failure		2
	Septic shock		1
	Septic pulmonary embolisms		8

### 3.2 Microbiological data

71.4 % of the cases had positive blood cultures. The etiology of IE was identified in 78.6 % (71.4 % through blood cultures and 7.2 % through a positive PCR test for *Coxiella burnetii* in explanted surgical material). In three cases (21.4 %), no microorganism was isolated despite repeated blood culture testing. The causative microorganisms were *Streptococcus* viridans group (4 cases), methicillin-sensitive *Staphylococcus aureus* (2 cases), *Staphylococcus epidermidis* (2 cases), *Coxiella burnetii* (1 case), *Haemophilus parainfluenzae* (1 case), and *Granulicatella adiacens* (1 case). Among the seven cases that underwent surgical intervention for PPVIE, six (85.7 %) had the same microorganism isolated from explanted material as in blood cultures, while in one case, no microorganism was isolated in the explanted material (PPVIE on a Biopulmonic™ valve due to *Streptococcus* viridans group, after antibiotic treatment, where large vegetations were described on the PPV during surgery). Microbiological findings are summarized in Table 2.

**Table 2:** Microbiological findings.

Positive blood culture		10
Positive culture of prosthesis surgically explanted		6 (85,7 %)
Etiology		
	Viridans streptococci	4
	*Staphylococcus aureus*	2
	Staphylococcus epidermidis	2
	Coxiella burnetti	1
	Haemophilus parainfluenzae	1
	Granulicatella adiacens	1
	Unknown	3

### 3.3 Echocardiographic data

All cases had both a pre-IE TTE and a TTE at the time of PPVIE diagnosis. In one case, neither images nor echocardiographic parameters from the PPVIE episode were available, and only a brief description of the findings was documented. TTE was reported as compatible with PPVIE in seven cases, with six showing vegetations and one showing leaflet rupture. No other cases exhibited leaflet rupture or perforation, paravalvular complications, or prosthetic dehiscence. The remaining seven TTEs were reported as non-diagnostic for IE (inconclusive or negative), yielding a TTE sensitivity of 50 % for PPVIE diagnosis.

**Fig. 1:**
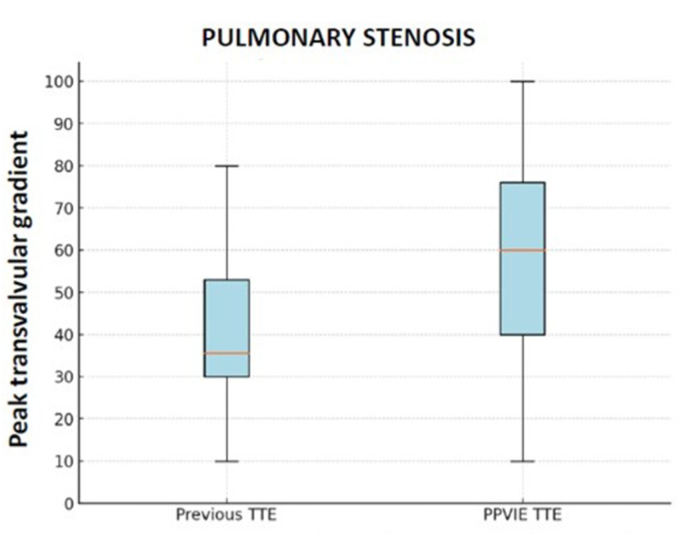
Comparison of peak transvalvular gradient (PTG) in the previous TTE and PPVIE TTE.

**Fig. 2:**
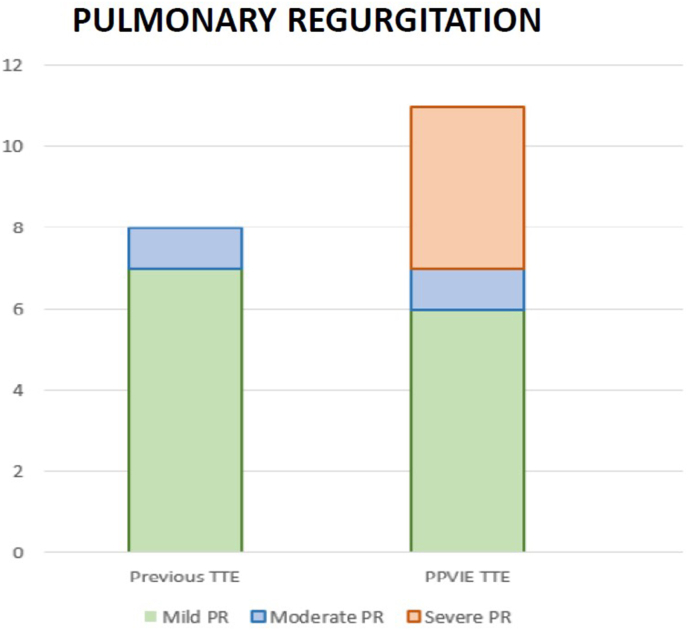
Comparison of PR in the previous TTE and PPVIE episode TTE.

The pre-IE TTE PTG was 35.5 mmHg (29.3–54.3), whereas the first TTE after suspected PPVIE diagnosis showed a PTG of 60.0 mmHg (40.0–78.0) (Fig. 1). The median difference between pre-IE PTG and PPVIE episode PTG was 20.0 mmHg (10.0–36.0). In the pre-IE TTE, no patient had severe PR, one had moderate PR, seven had mild PR, and six had no PR. At PPVIE diagnosis, four cases had severe PR, one had moderate PR, six had mild PR, and two had no PR (in one case, the PR severity was not documented in the report, and the corresponding images were unavailable) (Fig. 2).

Before the IE episode, prosthetic dysfunction was present in six cases (42.9 %), with five due to obstruction and one due to PR. At PPVIE diagnosis, 12 of the 13 patients (92.3 %) in whom PTG and PR severity were quantified had prosthetic dysfunction (seven due to stenosis, one due to PR, and four due to combined valve disease). The remaining patient did not have any echocardiographic images available, just a brief description of the echocardiographic findings, which did not include the PTG or description of PR severity. A detailed description of echocardiographic findings is provided in Table 3.

**Table 3:** Echocardiographic findings. RV: right ventricle. PA: pulmonary artery. PPVIE: endocarditis associated to prosthetic pulmonary valve and pulmonary conduit. TTE: transtorathic echocardiogram. PTG: peak transvalvular gradient. PR: pulmonary regurgitation. NA: not available.

	Type of RV-PA substrate	Time from prosthesis to PPVIE (years)	Etiology	Previous TTE			TTE in the PPVIE episode								Comparison	
				PTG (mmHg)	Grade of PR (0–3)	Prosthetic dysfunction	Result for PPVIE diagnosis	Vegetations	Leaflet rupture or perforation	Paravalvular complications	Prosthesis dehiscence	PTG (mmHg)	Grade of PR	Prosthetic dysfunction	PTG (mmHg)	Grade of PR
1	CONTEGRA™	0,6	Unknown	10	1	NO	COMPATIBLE	YES	NO	NO	NO	10	1	NO	=	=
2	CONTEGRA™	7.7	S. oralis	18	1	NO	INCONCLUSIVE	NO	NO	NO	NO	NA	NA	NA	NA	NA
3	CONTEGRA™	11,5	S. aureus	35	1	NO	COMPATIBLE	YES	NO	NO	NO	65	3	YES	+30	+2
4	BIOPULMONIC™	0,4	Unknown	30	2	YES	COMPATIBLE	YES	NO	NO	NO	24	2	YES	−6	=
5	MELODY™ (in Contegra™)	1,0	Unknown	36	0	NO	COMPATIBLE	YES	NO	NO	NO	80	0	YES	+44	=
6	MELODY™ (in homograft)	6,6	S. anginosus	37	1	NO	NEGATIVE	NO	NO	NO	NO	76	1	YES	+39	=
7	BIOPULMONIC™	0,2	S. epidermidis	30	0	NO	COMPATIBLE	YES	NO	NO	NO	48	1	YES	+18	+1
8	BIOPULMONIC™	4,3	S. mutans	50	1	YES	INCONCLUSIVE	NO	NO	NO	NO	70	1	YES	+20	=
9	CONTEGRA™	15,1	S. aureus	71	0	YES	INCONCLUSIVE	NO	NO	NO	NO	100	0	YES	+29	=
10	MELODY™ (in Contegra™)	2,2	C. burnetii	80	0	YES	COMPATIBLE	NO	YES	NO	NO	43	3	YES	−37	+3
11	MELODY™ (in homograft)	4,6	H. parainfluenzae	27	0	NO	NEGATIVE	NO	NO	NO	NO	40	1	YES	+13	+1
12	MELODY™ (in homograft)	8,1	S. mitins	54	1	YES	COMPATIBLE	YES	NO	NO	NO	80	3	YES	+36	+2
13	CONTEGRA™	7,4	G. adiacens	30	0	NO	INCONCLUSIVE	NO	NO	NO	NO	40	3	YES	+10	+3
14	PULMONARY BIOCONDUIT™	4,5	S. epidermidis	55	1	YES	INCONCLUSIVE	NO	NO	NO	NO	60	1	YES	+5	=

### 3.4 Complementary cardiac imaging data

In our series, the most frequently used additional imaging modalities were computarized tomography (CT) (10 cases), transesophageal echocardiography (TEE) (7), positron emission tomography – computerized tomography (PET-CT) (6), and single photon emission computerized tomography (1).

Among patients with non-diagnostic TTE for PPVIE, the additional imaging modalities included TEE in 71.4 % of cases (sensitivity 60 %), CT in 85.7 % (sensitivity 66.7 %), and PET-CT in 42.9 % (sensitivity 66.7 %). These techniques reclassified all patients with initially non-diagnostic TTE as definite IE.

### 3.5 Follow-up data

All patients were followed after the IE episode at our centre. The median follow-up duration was 3.4 years (2.2–5.3). Surgery was required in 50 % of cases. The indication for surgery was early IE in 1 case, heart failure in 2 cases, severe prosthetic dysfunction in 2 cases (one due to severe combined valve disease and the other due to severe stenosis) and uncontrolled infection (persistently positive blood cultures despite appropriate antibiotic treatment) in 2 cases. Median time between diagnosis and intervention was 15 days (12–25). No mortality was observed during the follow-up period.

## 4 Discussion

IE prevalence in CHD patients is known to be higher than in general population [2]. Moreover, many CHD are considered a predisposing factor for IE and are included as a minor criterion in the Duke criteria for IE [6]. It entails significant morbidity and mortality in this group of patients [11], which makes this condition a major concern for CHD specialists.

Many CHD primarily affect the right side of the heart, often requiring valvulation of the RVOT. In most cases, the material used is of biological origin and is subject to valve degeneration over time. Over the past few decades, advances in CHD management have significantly improved patient survival, particularly among those with complex conditions [10] and frequently these patients outlive their prosthesis. When this material deteriorates, replacement or an alternative approach for right ventricle-to-pulmonary artery continuity is necessary, either surgically or percutaneously. As a result, the number of interventions on the PV or RVOT has increased substantially, leading to a growing population of patients at risk of developing PPVIE.

In our series, Gram-positive cocci were the most common etiological agents of PPVIE, consistent with previous studies [5,[12], [13], [14]]. There were only two cases caused by *Staphylococcus aureus*, and only one required surgery, a proportion similar to that observed with other etiologies. The majority of cases occurred in males (78.6 %), which could be explained by the higher prevalence of CHD in the male population [10] and the potential antimicrobial effect of estrogens [15].

Regarding the PPVIE substrate, 35.7 % of cases involved Melody™ valves (21.4 % in Melody™ implanted in a homograft and 14.3 % in Melody™ implanted in Contegra™), while 35.7 % of cases occurred in Contegra™ conduits. Both prosthesis are derived from bovine jugular veins and, consistent with previous studies [14,16], have been associated with an increased risk of endocarditis.

As observed in our series and in previous studies [5,13], most cases of PPVIE occur late after PV intervention. In our cohort, the median time from prosthesis implantation to PPVIE development was 4.6 years (0.9–7.8 years), with three cases of early IE. These findings suggest that bacterial inoculation, in most cases, is not related to the implantation technique itself [17].

The literature regarding imaging techniques in PPVIE is scarce, and published studies report a limited number of cases. Miranda et al. [5] reported a series of 17 PPVIE cases, describing their clinical, microbiological, and echocardiographic characteristics. Venet et al. [12] analyzed a series of 66 suspected PPVIE cases (44 confirmed IE), evaluating the diagnostic performance of PET-CT.

Diagnosing this condition is challenging, and a high index of suspicion is required. Regarding clinical presentation, in our series, 64.3 % of cases presented with fever (≥38 °C), whereas in IE cases overall, this value is typically higher (77.7 % in the EURO-ENDO registry) [18]. Most cases had nonspecific accompanying symptoms (fatigue, arthromyalgia, headache, vomiting). Additionally, symptoms resulting from pulmonary embolic events can mimic a viral illness and may act as a confounding factor. In the EURO-ENDO registry [18], 25.3 % of cases presented with symptomatic septic embolisms at admission. In our series, despite 57.1 % of cases showing pulmonary septic embolisms on CT or FDG-PET, only one case presented with compatible symptoms (cough and pleuritic chest pain). Therefore, in CHD patients with a PPV or PC and fever without an obvious alternative origin, PPVIE should be ruled out.

**Image 1:**
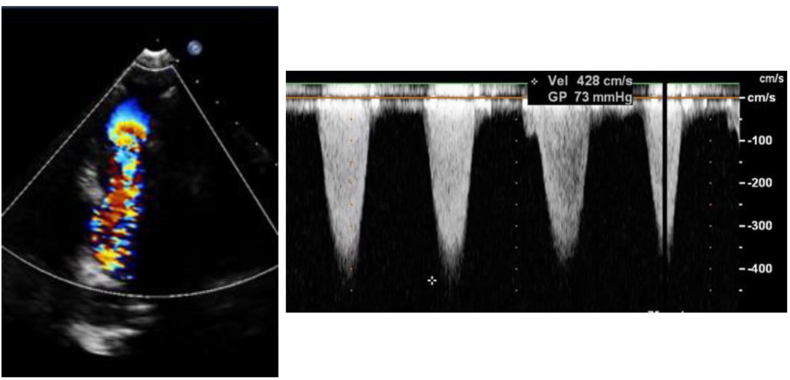
Color doppler and peak transvalvular gradient showing a severe stenosis in a surgically implanted Biopulmonic™ prosthesis in a patient with pulmonary atresia with intact ventricular septum.

**Image 2:**
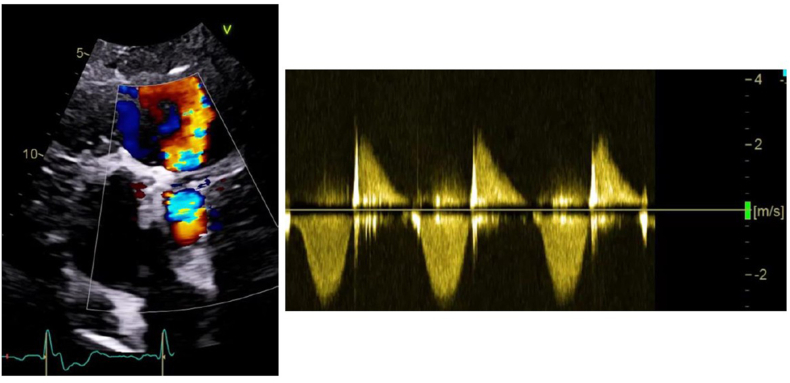
Color doppler and continuous doppler showing a severe pulmonary regurgitation in a Melody™ valve (in Contegra™) in a multioperated patient wit tetralogy of fallot.

TTE is the first-line imaging modality for suspected IE due to its accessibility and safety [4]. Its sensitivity in left-sided IE has been reported to be as high as 89.1 % in native valve cases within the EURO-ENDO registry, though slightly lower in prosthetic valve IE [18]. In PPVIE, TTE has limitations, and in our series, its sensitivity was only 50 %, slightly lower than the 62 % reported by Miranda et al. [5]. However, TTE provides information on prosthetic dysfunction, which, when combined with compatible clinical symptoms, may raise suspicion of IE. In our cohort, 92.3 % of cases presented with prosthetic dysfunction at PPVIE diagnosis, with 91.7 % showing stenosis and 41.7 % showing pulmonary regurgitation (33.3 % with combined valve disease) (Image 1, Image 2). Similar findings have been reported in other studies [5,12,14,19].

A new prosthetic dysfunction or acute worsening of a previously known prosthetic dysfunction (both regurgitation and/or stenosis) should be considered in the probability of IE diagnosis. In the case of PPVIE, the development of a new pulmonary stenosis or the worsening of a pre-existing stenosis is a more common mechanism of prosthetic dysfunction than pulmonary regurgitation. Although a new valvular stenosis is not included in the Duke criteria for the diagnosis of IE, some authors suggest that it should be considered a major criterion in PPVIE [20].

**Image 3:**
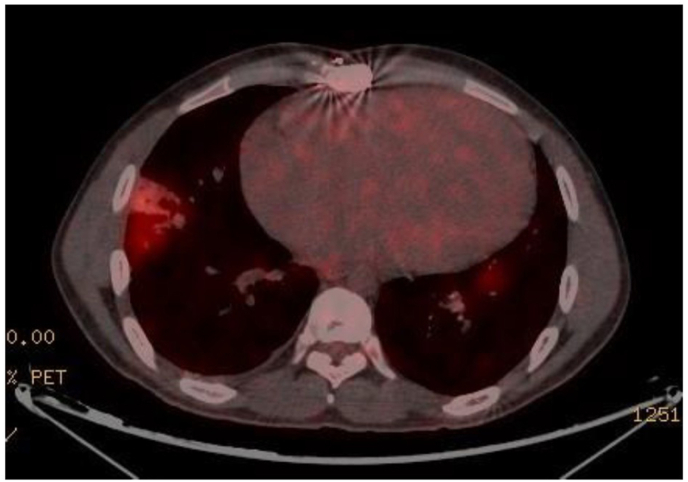
PET-CT showing a right pulmonary embolism.

Among the seven cases where TTE was not considered compatible with IE (non-diagnostic), in one case, echocardiographic parameters were unavailable, and in the remaining six, prosthetic dysfunction was present (five with stenosis and one with combined valve disease). Complementary imaging techniques reclassified all of these patients as definite IE, proving that prosthetic dysfunction on TTE is a marker that should prompt further study if PPVIE is suspected. Additionally, CT and PET-CT can diagnose pulmonary septic embolisms (Image 3) and detect paravalvular complications, which may be clinically relevant for decision-making.

Our study has some limitations. First, which is almost inherent to any PPVIE study, is the limited sample size of 14 cases, despite including data from over 10 years in a tertiary centre receiving CHD patients from other institutions. Another limitation is the lack of prosthetic dysfunction data in patients with PPV or PC without a PPVIE diagnosis. Thus, the specificity of prosthetic dysfunction for PPVIE diagnosis cannot be calculated. However, the presence of prosthetic dysfunction in 42.9 % of cases in the pre-IE TTE suggests that specificity may not be very high.

## 5 Conclusion

PPVIE cases have been relatively rare to date. However, with increasing survival rates among CHD patients and the growing number of PV interventions, the incidence of PPVIE is likely to rise in the future. This condition is challenging to diagnose using standard imaging techniques such as TTE. In the presence of compatible clinical symptoms, prosthetic dysfunction should raise suspicion of PPVIE and prompt further evaluation with advanced imaging modalities to confirm or rule out the diagnosis. Valvular stenosis is at least as common as pulmonary regurgitation in PPVIE patients, despite not being recognized as a criterion in the Duke classification.

## CRediT authorship contribution statement

**Andrés Cano Pérez:** Writing – review & editing, Writing – original draft, Visualization, Validation, Supervision, Software, Resources, Project administration, Methodology, Investigation, Formal analysis, Data curation, Conceptualization. **Larraitz Orive Melero:** Visualization, Validation, Supervision, Formal analysis, Data curation, Conceptualization. **Jose Félix Larrea Egurbide:** Validation, Supervision, Formal analysis, Conceptualization. **Jagoba Larrazábal López:** Visualization, Validation, Methodology, Formal analysis, Conceptualization. **Luis Fernández González:** Visualization, Validation, Investigation, Data curation, Conceptualization. **Roberto Blanco Mata:** Visualization, Validation, Supervision, Formal analysis, Conceptualization. **Josune Arriola Meabe:** Visualization, Validation, Investigation, Data curation, Conceptualization. **Leire Artiñano Mendizábal:** Validation, Supervision, Formal analysis, Conceptualization. **Ane Josune Goikoetxea Agirre:** Visualization, Validation, Supervision, Investigation, Data curation, Conceptualization. **María José Blanco Vidal:** Validation, Supervision, Software, Formal analysis, Conceptualization. **Javier Ayala Curiel:** Visualization, Validation, Supervision, Formal analysis, Conceptualization. **Pedro María Montes Orbe:** Visualization, Validation, Supervision, Formal analysis, Conceptualization.

## Disclosures

None of the authors receive funding from the industry, neither for this work nor for any other.

## Funding

None.

## Declaration of competing interest

The authors declare that they have no known competing financial interests or personal relationships that could have appeared to influence the work reported in this paper.
